# *Ostreopsis* cf. *ovata* (Dinophyceae) Molecular Phylogeny, Morphology, and Detection of Ovatoxins in Strains and Field Samples from Brazil

**DOI:** 10.3390/toxins12020070

**Published:** 2020-01-22

**Authors:** Silvia M. Nascimento, Raquel A. F. Neves, Gabriela A. L. De’Carli, Geovanna T. Borsato, Rodrigo A. F. da Silva, Guilherme A. Melo, Agatha M. de Morais, Thais C. Cockell, Santiago Fraga, Adriana D. Menezes-Salgueiro, Luiz L. Mafra, Philipp Hess, Fabiano Salgueiro

**Affiliations:** 1 Laboratório de Microalgas Marinhas, Universidade Federal do Estado do Rio de Janeiro (UNIRIO), Av. Pasteur, 458, 314-B, Rio de Janeiro, RJ 22290-240, Brazil; raquel.neves@unirio.br (R.A.F.N.); geoborsato@hotmail.com (G.T.B.); rodrigoalmeidafs@yahoo.com.br (R.A.F.d.S.); agathamiralha@yahoo.com.br (A.M.d.M.); thaiscockell@hotmail.com (T.C.C.); 2 Laboratório de Biodiversidade e Evolução Molecular, Universidade Federal do Estado do Rio de Janeiro (UNIRIO), Av. Pasteur, 458, 512, Rio de Janeiro, RJ 22290-240, Brazil; guimelo93@gmail.com; 3 Centro Oceanográfico de Vigo, Instituto Español de Oceanografía (IEO), Subida a Radio Faro 50, 36390 Vigo, Spain; santi.fraga.ieo.vigo@gmail.com; 4 Laboratório de Biotecnologia Vegetal, Instituto Federal de Educação, Ciência e Tecnologia do Rio de Janeiro, Rua Senador Furtado, 121, 112. Maracanã, Rio de Janeiro, RJ 20270-021, Brazil; adriana.salgueiro@ifrj.edu.br; 5 Centro de Estudos do Mar, Universidade Federal do Paraná, Cx. Postal 61, Pontal do Paraná, PR 83255-976, Brazil; luiz.mafra@ufpr.br; 6 IFREMER, Laboratoire Phycotoxines, Rue de l’Ile d’Yeu, 44311 Nantes, France; Philipp.Hess@ifremer.fr

**Keywords:** benthic dinoflagellates, harmful algal blooms, ovatoxins, taxonomy, morphometry

## Abstract

Recurrent blooms of *Ostreopsis* cf. *ovata* have been reported in Brazil and the Mediterranean Sea with associated ecological, and in the latter case, health impacts. Molecular data based on the D1–D3 and D8–D10 regions of the LSU rDNA and ITS loci, and the morphology of *O*. cf. *ovata* isolates and field populations from locations along the Brazilian tropical and subtropical coastal regions and three oceanic islands are presented. Additional ITS sequences from three single cells from the tropical coast are provided. Toxin profiles and quantities of PLTX and their analogues; OVTXs; contained in cells from two clonal cultures and two field blooms from Rio de Janeiro were investigated. Morphology was examined using both light and epifluorescence microscopy. Morphometric analysis of different strains and field populations from diverse locations were compared. Molecular analysis showed that six of the seven sequences grouped at the large “Atlantic/Mediterranean/Pacific” sub-clade, while one sequence branched in a sister clade with sequences from Madeira Island and Greece. The toxin profile of strains and bloom field samples from Rio de Janeiro were dominated by OVTX-a and -b, with total cell quotas (31.3 and 39.3 pg cell^−1^) in the range of that previously reported for strains of *O*. cf. *ovata*.

## 1. Introduction

*Ostreopsis* Johs. Schmidt is a genus of benthic dinoflagellate that has been extensively studied in recent years due to the impacts caused by their recurrent toxic blooms to human health and marine ecosystems. The genus was first identified in tropical areas [1,2,3], and later studies showed the presence of *Ostreopsis* species in temperate regions [4]. To date, *Ostreopsis* comprises eleven species, namely *O. siamensis* Johs. Schmidt [1], *O. lenticularis* Y. Fukuyo [2], *O. ovata* Y. Fukuyo [2], *O. heptagona* D.R. Norris, J.W. Bomber & Balech [5], *O. mascarenensis* Quod [6], *O. labens* M.A. Faust & S.L. Morton [7], *O. belizeana* M.A. Faust, *O. caribbeana* M.A. Faust, *O. marina* M.A. Faust [3], *O. fattorussoi* Accoroni, Romagnoli & Totti [8], and *O. rhodesae* Verma, Hoppenrath & S.A. Murray [9].

The identification of the *Ostreopsis* species based solely on morphology is extremely difficult [10,11]. The plate pattern is largely similar among species and broadly fits the description of the type species, *Ostreopsis siamensis*, except for *Ostreopsis heptagona* [10,12,13]. The characters used to delineate species, such as variations in cell size, outline, and some slight differences on the shape of certain thecal plates [3,10,13] have been shown to vary within a given species (e.g., Penna et al. [10]). Moreover, only the two most recent *Ostreopsis* species descriptions include molecular sequence data and a genotype assignment with the morphological description [8,9].

The ambiguities in defining the morphological characters to resolve species of *Ostreopsis* have led to several attempts for the revision of *Ostreopsis* species using molecular data [11]. Many studies have used sequences of ITS and 5.8S regions and/or the D1–D3 and/or D8–D10 domains of the LSU of the rDNA, usually in combination with morphological observations to clarify species delineations [9,10,12,14,15,16]. While it was possible to ascertain genetic differences, the plasticity in morphology prevented the determination of clear morphospecies [11], and different genetic entities (i.e., “genotypes”) were named *Ostreopsis* sp. 1-6 by Sato et al. [14] and *Ostreopsis* sp. 7 by Tawong et al. [15]. Recently, Chomérat et al. [16] re-investigated the presence of *O. lenticularis* in its type locality, i.e., French Polynesia, and taxonomically assigned the genotype *Ostreopsis* sp. 5 to *O. lenticularis*, and indicated that *Ostreopsis* sp. 6 may correspond to the originally described *O. siamensis*.

**Figure 1:**
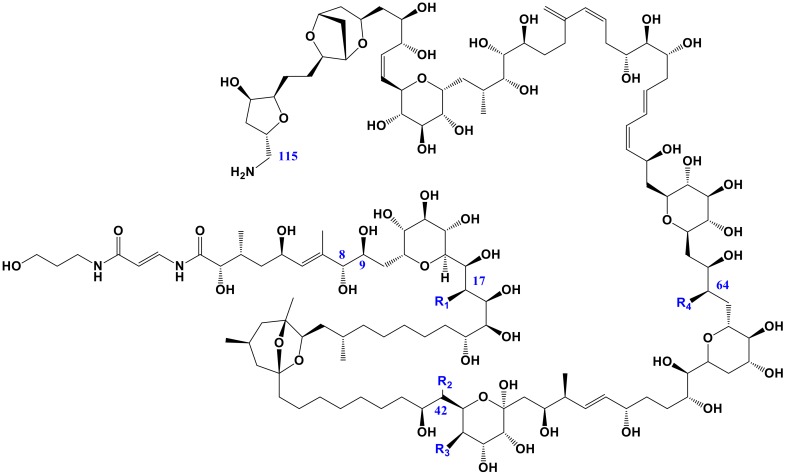
Planar structure of palytoxin (PLTX) and analogues. In PLTX: R1 = OH, R2 = H, R3 = OH, R4 = OH and in OVTX-a: R1 = H, R2 = OH, R3 = H, R4 = H. Reproduced with permission from Rossini and Hess [28], Phycotoxins: chemistry, mechanism of action and shellfish poisoning; published by Springer Nature: Basel, Switzerland, 2010.

Some species of *Ostreopsis* produce potent toxins. The study by Gleibs and Mebs [17] was the first to show that palytoxin and analogues (Figure 1) are not solely found in the zoanthids *Palythoa* spp. but also in many other organisms in tropical marine food webs. In particular, the finding of palytoxin or analogues in mussels and starfish suggested that a different organism than *Palythoa* spp. may exist. Subsequently, Ukena et al. [18] were able to show that analogues of palytoxin, i.e., ostreocins, were produced by *O. siamensis*, i.e., a dinoflagellate (contrary to palytoxin itself which is produced by *Palythoa* spp.). Subsequently, a number of analogues referred to as ovatoxins (OVTXs), have been described to be produced by *O*. cf. *ovata*. Aerolized palytoxin can induce severe respiratory distress [19] and putative PLTX is implicated in the fatal disease clupeotoxism [20]. Recurrent blooms of *O*. cf. *ovata* have been reported in the Mediterranean Sea where the exposure to marine aerosols has caused harmful effects to human health [21]. Symptoms of human intoxication included fever, bronchoconstriction with mild dyspnea, wheezing, conjunctivitis, and skin irritation. A recent study also outlined the similarity of symptoms in patients exposed to *Palythoa* spp. from aquariums and those exposed to *Ostreopsis* blooms [22]. Blooms of this species have also been associated to massive mortalities of marine invertebrates in the Mediterranean Sea [23,24], Brazil [25,26], and New Zealand [27].

In Brazil, blooms of *O*. cf. *ovata* are common along the Rio de Janeiro coast at Arraial do Cabo and Armação dos Búzios, where the species forms a brownish biofilm covering macroalgae (Nascimento et al., data not published). Massive blooms of *O.* cf. *ovata* have been observed at the remote equatorial oceanic island of St. Paul’s Rocks [29] and at the coastal subtropical Currais Archipelago [30]. High *O*. cf. *ovata* abundances were also found at the Brazilian tropical coast in Bahia, although no biofilm was observed [31]. The current study reports molecular data based on the D1–D3 and D8–D10 regions of the LSU rDNA and ITS loci and the morphology of *O*. cf. *ovata* isolates and field populations from locations along the Brazilian tropical and subtropical coastal regions and three oceanic islands. Additional ITS sequences from three single cells sampled from the tropical coast are provided. Toxin profiles and quantities of PLTX and analogues produced by two clonal cultures and two field blooms from Rio de Janeiro were investigated.

## 2. Results

### 2.1. Morphology

**Figure 2:**
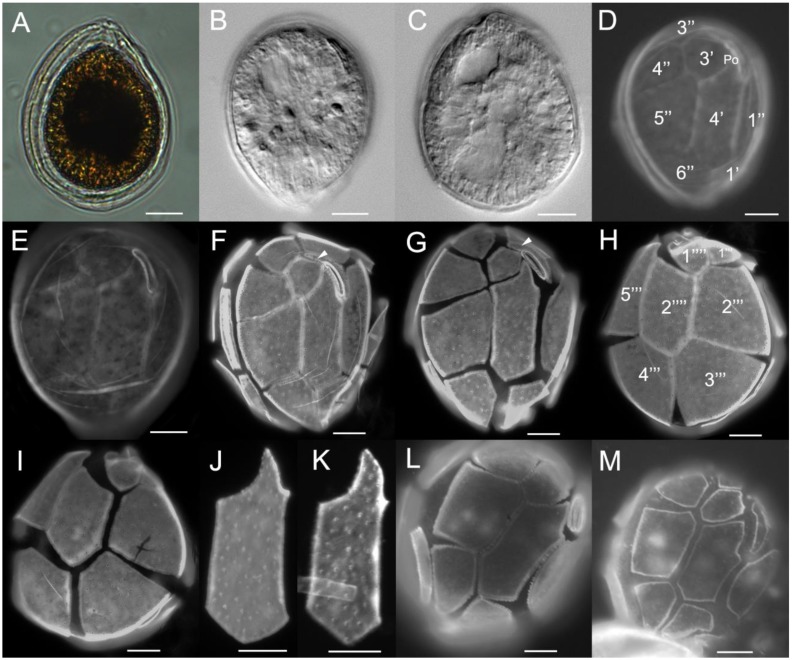
Light (**A**–**C**) and epifluorescence (**D**–**M**) micrographs of cells of *O*. cf. *ovata* strain UNR-10 from Rio Grande do Norte, Brazil (**A**–**K**), and strain VGO614 from Madeira Island, Portugal (**L**–**M**): (**A**) broadly oval cell shape; (**B**–**C**) elongated chloroplasts and the posterior nucleus are visible; (**D**–**G**) apical view, note the variable size of the suture between plates 3′ and 5” and variable shape of plate 4′, plate 2′ extends between plates 2” and 3′ (**F**–**G**, arrow heads); (**H**–**I**) antapical view; (**J**–**K**) plate 4′ variable shape; (**L**–**M**) apical view of two cells from strain VGO614. Scale bars: 10 µm.

**Figure 3:**
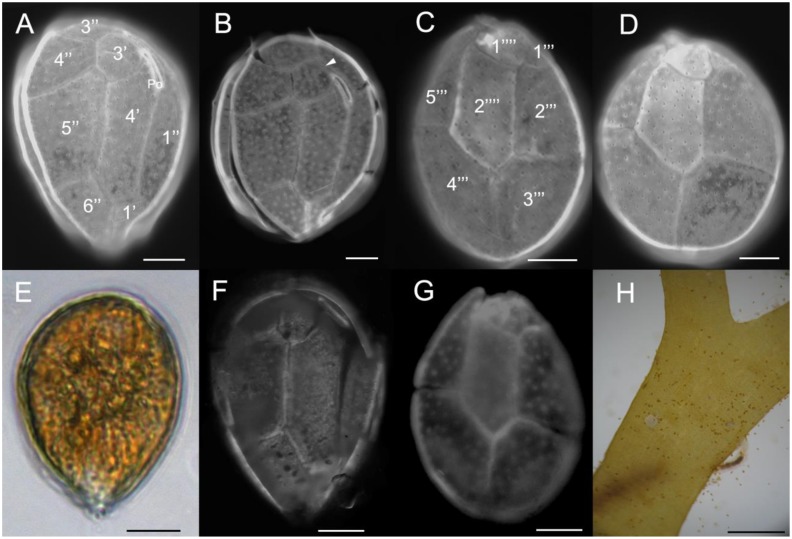
Epifluorescence (**A**–**D**, **F**–**G**) and light (**E**, **H**) micrographs of cells of *O*. cf. *ovata* strain UNR-05 from Rio de Janeiro, Brazil (**A**–**E**); field cells from Forte, Bahia (**F**–**G**); and from a bloom at Rio de Janeiro (H): (**A**–**B**, **F**) apical view; (**C**–**D**, **G**) antapical view; (**E**) live cell; (**H**) field live cells associated to the macroalgae *Canistrocarpus cervicornis* during a bloom at Arraial do Cabo, Rio de Janeiro, Brazil. Scale bars: (**A**–**G**): 10 µm; H: 1000 µm.

*Ostreopsis* cf. *ovata* cells from strains UNR-03, UNR-05, UNR-10 and UNR-60 were tear-shaped, oval, or broadly oval in apical/antapical view (Figure 2A–I and Figure 3A–G ). Cells of strain UNR-10 were broadly oval (Figure 2). Cells were highly compressed antero-posteriorly. The dorsoventral diameter (DV) of strain UNR-05 varied between 29.8 and 61.4 µm (mean ± standard deviation: 45.0 ± 6.4 µm, *n* = 100), width (W) ranged from 21.1 to 45.2 µm (mean 32.5 ± 5.1 µm, *n* = 100) and the mean DV/W ratio was 1.4 ± 0.1 (1.2–1.7). Among cells of this strain, dimensions of plate 4′ varied between 30.5 and 40.8 µm in depth (mean 34.1 ± 3.4 µm, *n* = 17) and 7.9 and 13.4 µm in width (mean 10.9 ± 1.4 µm, *n* = 17). For cells of strain UNR-10, the DV diameter ranged from 47.4 to 64.0 µm (53.7 ± 5.7 µm, *n* = 7) and W from 37.4 to 57. 7 µm (42.3 ± 7.8 µm, *n* = 6). Cells presenting a small suture between plates 3′ and 5″ or just touching in a point were observed in cultures of strains UNR-05 (not shown) and UNR-10 (Figure 2F–G). Cells from strain UNR-10 seemed wider than cells from strains UNR-03 and UNR-05, although no statistical test could be employed because cultures of strain UNR-10 were lost preventing further morphometric analysis.

The thecal surface was smooth and covered with scattered pores of a single class visible in light microscopy (Figure 2F–I and Figure 3A–D). The thecal plate pattern was APC, 4′, 6″, 6c, ?s, 5‴, 2″″, and thecal plates were clearly visible with epifluorescence microscopy (Figure 2D–I and Figure 3A–D,F–G). The apical pore complex (APC) consisted of a narrow, elongated and slightly curved Po plate bearing a slit and two rows of pores (Figure 2F and Figure 3B). There were four apical plates, considering the system by Besada et al. [32]. The fourth apical plate (4′) was located at the center of the epitheca and was elongated and hexagonal (Figure 2D–G and Figure 3A–B). The shape of plate 4′ was variable and nearly heptagonal plate 4′ were also observed (Figure 2J–K). The second apical plate (2′) which was located below the APC, was narrow and elongated, extending between plates 2″ and 3′ (Figure 2F-G and Figure 3B), and the third apical plate (3′) was irregularly pentagonal (Figure 2D–G and Figure 3A–B). In the observed cells of strain VGO614, that branched in the same sub-clade as strain UNR-10 (see Section 2.2) plate 4″ was hexagonal and plates 3′ and 2″ were in contact as plate 2′ did not have an extension (Figure 2L–M). There were six precingular plates. Plates 1″, 2″, 3″, 4″, and 6″ were irregularly quadrangular, while plate 5″ was mostly pentagonal or quadrangular as in Figure 2G, and the largest of the precingular series (Figure 2D–G and Figure 3A–B).

In the hypotheca, there were five postcingular plates. Plates 2‴, 3‴ and 4‴ were the largest of this series (Figure 2H–I and Figure 3C–D,G). Plate 1‴ was smaller than the other postcingular plates. Plates 2‴, 3‴ and 4‴ were quadrangular and plate 5‴ was oblong and irregularly triangular (Figure 2H–I and Figure 3C–D,G). In the antapical series, the first antapical plate (1″″) was small and triangular while plate 2″″ presented an elongated pentagonal shape (Figure 2H–I and Figure 3C–D). Cells presented many golden brown chloroplasts (Figure 3E).

Field-sampled cells isolated from Forte, Bahia (Figure 3F–G) for genetic analysis and from the bloom samples at Rio de Janeiro also presented the morphology in agreement with that of *O*. cf. *ovata*. The three field-sampled cells isolated from Forte, Bahia presented the following dimensions (DV, W): 45, 28 μm; 58, 37 μm and 56, 44 μm. During the bloom at Arraial do Cabo, many *O*. cf. *ovata* cells were observed associated to the macroalgae *Canistrocarpus cervicornis* by threads of mucilage (Figure 3H).

**Figure 4:**
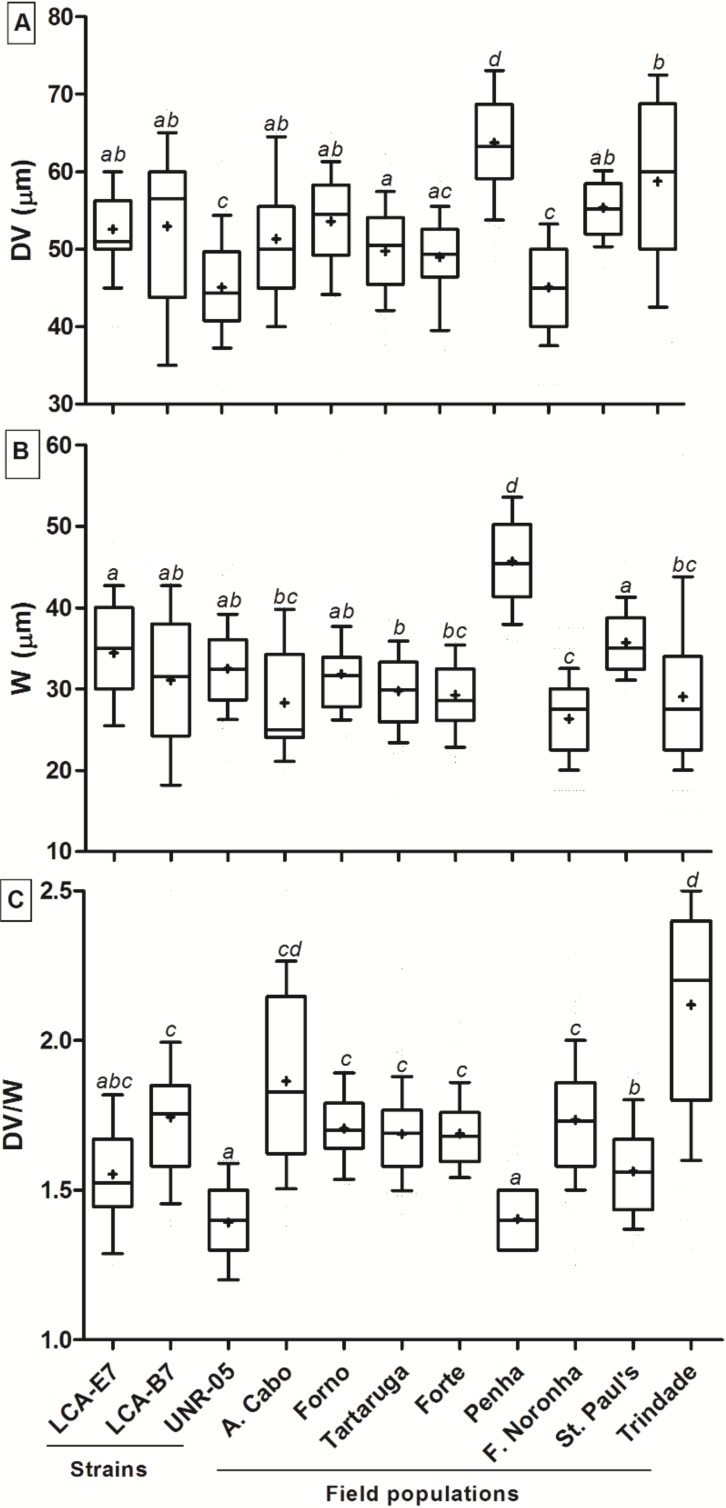
Morphometric analysis: (**A**) Dorso-ventral (DV) diameter, (**B**) width (W) and (**C**) DV/W ratio of cultivated (LCA-E7, LCA-B7, UNR-05) and field cells of *O*. cf. *ovata* from coastal locations at Rio de Janeiro state (A. Cabo, Forno, Tartaruga) and Bahia (Forte, Penha) and from the oceanic islands of Fernando de Noronha, St. Pauls´s Rocks and Trindade, Brazil. Median (-), mean (+), and whiskers (10–90 percentile) are shown. Different letters indicate statistically significant differences (multiple comparisons, *p* < 0.03).

Dorso-ventral diameter (DV), width (W), and DV/W ratio of cells from cultivated strains and field populations collected at five sites along the Brazilian coast and three oceanic islands were in the range reported in the literature for *O.* cf. *ovata* (Table 1, see Figure S1 for DV variability). *Ostreopsis* cf. *ovata* cells from cultured strains and field populations showed significant variations in the values of DV (Kuskal-Wallis, H_(10,965)_ = 401.41, *p* < 0.001), W (Kuskal-Wallis, H_(10,965)_ = 380.26, *p* < 0.001) and DV/W (Kuskal-Wallis, H_(10,965)_ = 523.83, *p* < 0.001) (Figure 4A–C). Cells of strain UNR-05 and from Fernando de Noronha oceanic island had significantly lower DV diameter (multiple comparisons, *p* ≤ 0.05), while cells from Penha, Bahia showed significantly higher DV diameter (multiple comparisons, *p* ≤ 0.0004) in comparison to the other strains and field populations (Figure 4A). Cells from Fernando de Noronha island were significantly narrower (multiple comparisons, *p* ≤ 0.02) than cultured strains and other field populations, while cells from Penha were significantly larger (multiple comparisons, *p* ≤ 0.0001) (Figure 4B). DV/W ratio of cells from strain UNR-05 and Penha was significantly lower (multiple comparisons, *p* ≤ 0.0001), while cells from Trindade oceanic island showed significantly higher DV/W values (multiple comparisons, *p* ≤ 0.01) in comparison to cultured strains and other field populations (Figure 4C). At Penha, cells presented a larger variability in shape, ranging from tear- and narrow- shaped to large- and round- shaped. At that location, cell size (both DV and W) was significantly larger (multiple comparisons, *p* < 0.001; Figure 4) and more variable (wider range) than that observed at other sites in Brazil and elsewhere (Table 1). Larger cells, with a DV diameter ranging between 75–85 µm, which is higher than the DV frequently reported for *O.* cf. *ovata* (see Table 1, although David et al. [12] reported DV values of up to 84 µm) were found to represent less than 10% of total cells from Penha.

### 2.2. Molecular Phylogeny

**Figure 5:**
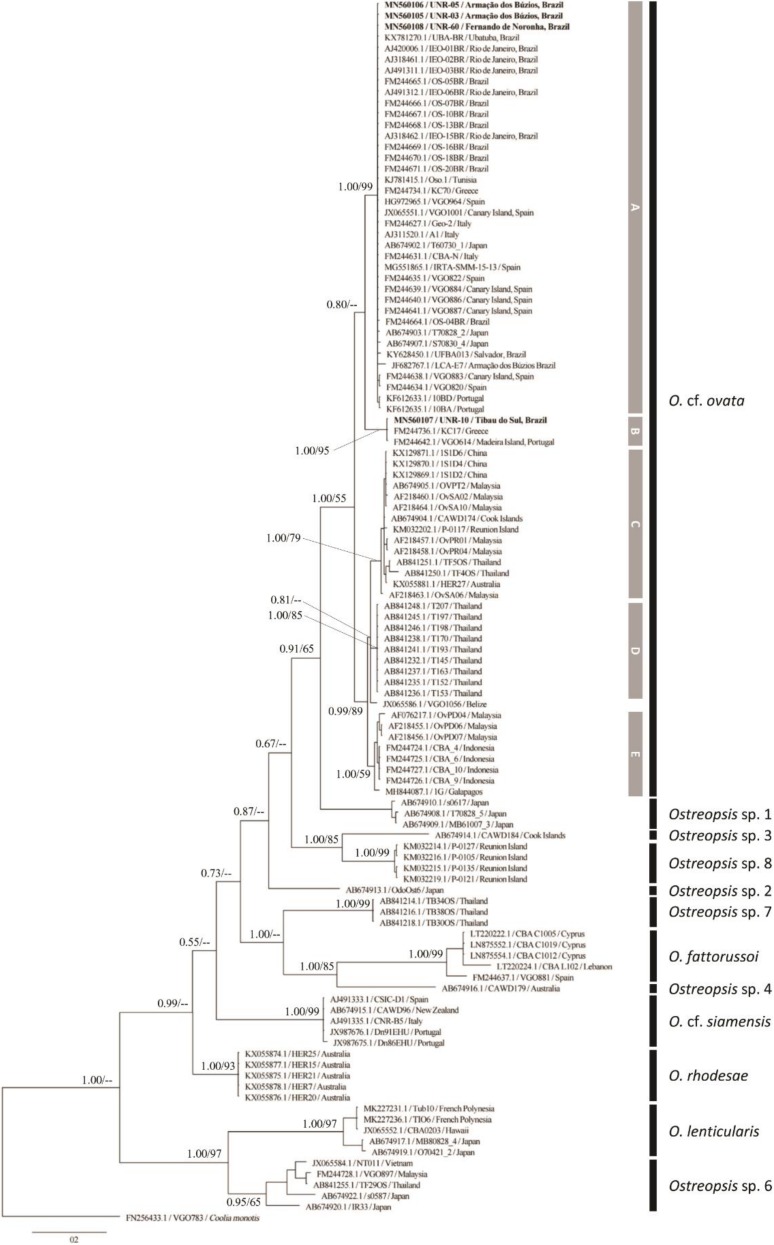
Bayesian Inference phylogenetic tree based on ITS rDNA sequences of several *Ostreopsis* strains: Operational taxonomic units (OTUs) are identified by GenBank accession number\strain name\locality. Numbers at nodes represent posterior probability from BI and bootstrap values from ML analyses, respectively (cut-off = 50% for both analyses). New sequences from this study are displayed in bold (UNR-03, UNR-05, UNR-10 and UNR-60).

**Figure 6:**
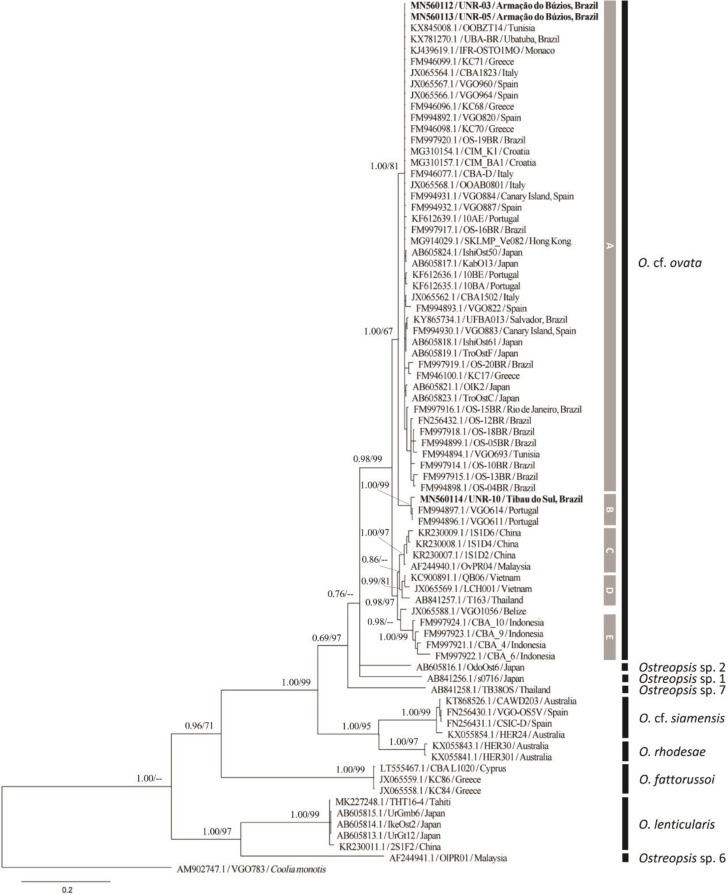
Bayesian Inference phylogenetic tree based on LSU rDNA D1–D3 sequences of several *Ostreopsis* strains: Operational taxonomic units (OTUs) are identified by GenBank accession number\strain name\locality. Numbers at nodes represent posterior probability from BI and bootstrap values from ML analyses, respectively (cut-off = 50% for both analyses). New sequences from this study are displayed in bold (UNR-03, UNR-05 and UNR-10).

**Figure 7:**
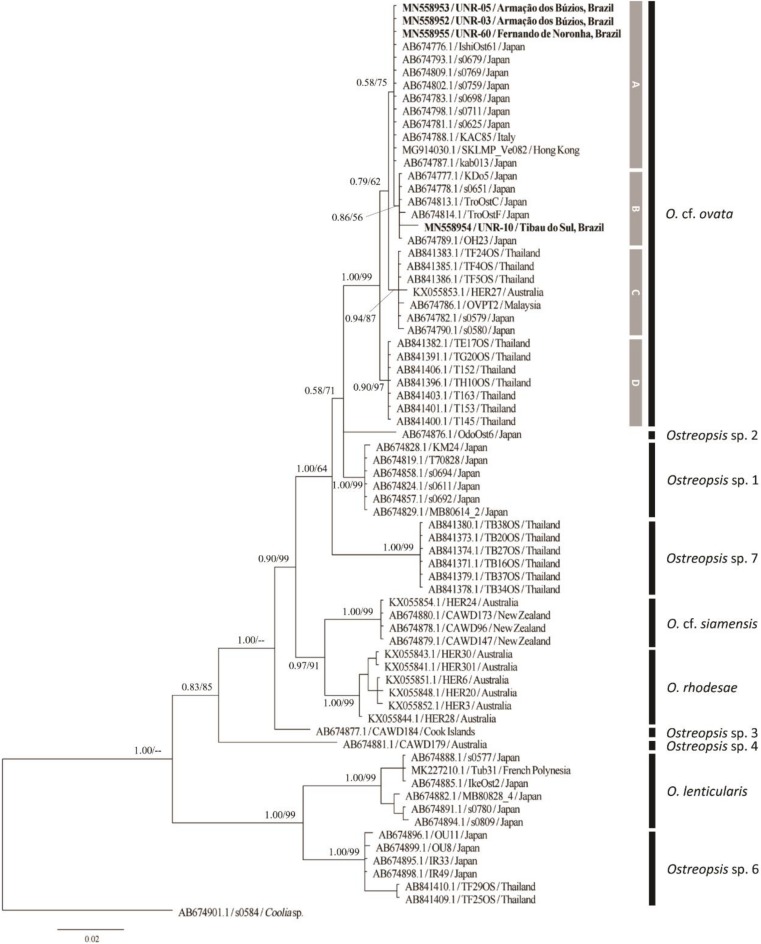
Bayesian Inference phylogenetic tree based on LSU rDNA D8–D10 sequences of several *Ostreopsis* strains: Operational taxonomic units (OTUs) are identified by GenBank accession number\strain name\locality. Numbers at nodes represent posterior probability from BI and bootstrap values from ML analyses, respectively (cut-off = 50% for both analyses). New sequences from this study are displayed in bold (UNR-03, UNR-05, UNR-10 and UNR-60).

Molecular analysis confirmed the identity of all the strains and field-sampled cells analyzed in the current study as *Ostreopsis* cf. *ovata*. Analyses of maximum likelihood and Bayesian inference yielded phylogenetic trees with similar topologies. The relationships among *Ostreopsis* species and *Ostreopsis* cf. *ovata* clades were congruent with those observed in the literature [8,9,14,15,16,33]. Hence, only the topologies of the BI phylogenetic trees are presented in the manuscript (Figure 5, Figure 6 and Figure 7 for ITS, D1–D3 and D8–D10, respectively).

#### 2.2.1. ITS rDNA Phylogeny

The phylogenetic analysis inferred from ITS region encompassed four new sequences generated in the present study from strains isolated from the Brazilian coast (South Atlantic Ocean). The final alignment comprised 112 sequences and 418 aligned nucleotides. The phylogenetic tree based on ITS sequences presented well supported clades that represented the 12 species/phylotypes within the genus: *O. fattorussoi*, *O. lenticularis*, *O*. cf. *ovata*, *O.* cf. *siamensis*, *O. rhodesae, Ostreopsis* sp. 1, *Ostreopsis* sp. 2, *Ostreopsis* sp. 3, *Ostreopsis* sp. 4, *Ostreopsis* sp. 6, *Ostreopsis* sp. 7, and *Ostreopsis* sp. 8 (Figure 5).

Five subclades of *Ostreopsis* cf. *ovata* were recognized in the ITS tree (subclades A to E, Figure 5). Strains UNR-03, UNR-05 and UNR-60 grouped in the *O*. cf. *ovata* subclade A together with sequences of strains from the Mediterranean Sea, the NE Atlantic (Canary Island and Portugal) as well as the SE Atlantic (Rio de Janeiro, Bahia and São Paulo in Brazil) and Japan. This is the Mediterranean/Atlantic/Pacific (AMP) clade as described by Zhang et al. [33] and Tibiriçá et al. [30]. Its sister subclade (B) contained three sequences, including strain UNR-10 isolated from the Brazilian Northeast coast, strain VGO614 from Madeira Island (Portugal) and strain KC17 from the Mediterranean Sea (Greece) (Figure 5). Subclades C, D, and E were composed exclusively of strains from the Indo-Pacific region. The subclade C (called South China Sea by Penna et al. [34]) was composed of strains from Australia, Cook Islands, Malaysia, Thailand and Reunion Island. Subclade D included strains exclusively from Thailand while subclade E contained strains from Indonesia, Malaysia and Galapagos Islands (Figure 5).

The three ITS sequences obtained from the field-sampled cells from Bahia, Brazil were excluded from the final phylogenetic threes because they were slightly shorter (~265 nt). However, BLAST (Basic Local Alignment Search Tool) and phylogenetic analyses performed with these sequences confirmed their identity as *Ostreopsis* cf. *ovata* (subclade A).

#### 2.2.2. LSU rDNA D1–D3 Phylogeny

Phylogenetic reconstructions based on D1–D3 region included three new sequences from the current study and other 75 sequences retrieved from GenBank, totaling 78 OTUs and 720 aligned nucleotides. The overall tree topology was similar to that observed with ITS and D8–D10 sequences in the current study, and with D1–D3 sequences by other authors [8,9,33] (Figure 6). There are no D1–D3 sequences available for *Ostreopsis* sp. 3, *Ostreopsis* sp. 4 and *Ostreopsis* sp. 8. Concerning the *O.* cf. *ovata* clade, the five subclades (A to E) observed in the ITS analysis (Figure 5) were recognized in the LSU D1–D3 phylogenetic tree (Figure 6). Strains UNR-03 and UNR-05 grouped into *O.* cf. *ovata* subclade A together with sequences of strains from the Mediterranean Sea, the Atlantic coast (Europe and Brazil), as well as Japan and Hong Kong. As previously observed in the analyses based on ITS loci, the strain UNR-10 branched in *O.* cf. *ovata* subclade B (Figure 6) together with two sequences from strains isolated from the Madeira Island (VGO611 and VGO614).

#### 2.2.3. LSU rDNA D8–D10 Phylogeny

The D8–D10 dataset included four new sequences from the present study and 71 sequences from GenBank, totaling 75 OTUs and 821 aligned nucleotides. Phylogenetic analyses included sequences of *O.* cf. *ovata*, *O*. *lenticularis*, *O.* cf. *siamensis*, *O. rhodesae, Ostreopsis* sp. 1, *Ostreopsis* sp. 2, *Ostreopsis* sp. 3, *Ostreopsis* sp. 4, *Ostreopsis* sp. 6 and *Ostreopsis* sp. 7 (Figure 7). Sequences of *O*. *fattorussoi* and *Ostreopsis* sp. 8 were not included in this analysis since they were not available in GenBank. Ten main clades were recovered for the *Ostreopsis* genus, the same observed by Chomérat et al. [16] when D8–D10 sequences were considered. Four subclades of *Ostreopsis* cf. *ovata* were recognized in the LSU rDNA D8–D10 tree (subclades A to D, Figure 7). The subclade E (observed in the ITS and D1–D3 trees) was not observed in the D8–D10 tree since there were no sequences from this lineage available in GenBank. Strains UNR-03, UNR-05 and UNR-60 from Brazil grouped in the *O.* cf. *ovata* subclade A together with sequences of strains isolated from Italy, Hong Kong and Japan. The fourth strain from Brazil, UNR-10, grouped in the *O.* cf. *ovata* subclade B together with sequences of strains from Japan.

### 2.3. Toxin Content and Profile

The toxin profile of both *O.* cf. *ovata* strains isolated from Rio de Janeiro and bloom field samples were dominated by OVTX-a followed by OVTX-b, whereas OVTX-c, -d and -e were found as minor components (Table 2). OVTX-a and -b represented on average 63.1% and 31.7%, respectively, of the total ovatoxin content for both strains and bloom field samples. The intracellular toxin content (cell quota) of strains UNR-03 and UNR-05 was similar, of 20.9 and 20.0 pg OVTX-a cell^−1^, 14.3 and 9.3 pg OVTX-b cell^−1^ (Table 2). OVTX-c, -d, and -e were minor components of the toxin profile with OVTX-e representing 4.6% of total OVTX content in bloom samples and only 0.3% in cultured strains. OVTX-f was not detected in any sample.

## 3. Discussion

### 3.1. Morphology

Almost all *Ostreopsis* species have a thecal plate pattern fitting with the original description of *O. siamensis* and therefore plate pattern cannot be used to unambiguously differentiate species within this genus. In the current study, *O.* cf. *ovata* cells with a small suture between plates 3′ and 5″ or just touching in a point were observed in cultures of strains UNR-05 and UNR-10, similar to cells depicted by Tibiriçá et al. [30] in their Figure 4B,F. The shape of plate 4′ was quite variable at the suture between this plate and plate 3′ (Figure 2F–G,J–K). In some cells of strain UNR-10 plate 4″ presented a curved suture between plates 3′ and 4′ (Figure 2J–K), a characteristic proposed as a distinguishing feature for *O. fattorussoi*, but then proved to be variable, as discussed by Tibiriçá et al. [30]. Moreover, in these cells of strain UNR-10 (Figure 2E–F) plate 2′ extended between plates 2″ and 3′, being longer than the APC plate, as already observed for other specimens of the *O*. cf. *ovata* phylogenetic clade by Penna et al. [10], Zhang et al. [33] and Tibiriçá et al. [30]. In the observed cells of strain VGO614 plate 2′ did not extend between plates 2″ and 3′. In *O. rhodesae* and *O. fattorussoi* the elongated plate 2′ is twice as long as the APC plate [8,9].

It is recognized that *O.* cf. *ovata* cell shape and size may exhibit great variability and overlap with other species in the genus [10,12,33]. In the current study, cell dimensions among various *O.* cf. *ovata* strains and field populations from diverse sites showed significant differences that may be caused by differences in growth phases, or by the influence of different environmental conditions. Accoroni et al. [39] showed that cells in the decline phase of a bloom had a significantly longer DV than those in both the initial and in the proliferation phases. Moreover, significantly lower DV were found in sheltered sites compared with exposed ones suggesting that turbulence can affect *O.* cf. *ovata* cell size [39]. In the current study, 10% of the population occurring at Penha presented DV values of 75–85 µm. Both DV and W values were significantly higher at Penha compared to the three cultured strains and the other field populations and therefore it is possible that these cells represent another *Ostreopsis* species that likely co-occur with *O.* cf. *ovata* at this site, however this requires confirmation by molecular analysis.

### 3.2. Molecular Phylogeny

*Ostreopsis* cf. *ovata* have been shown to be widely dispersed from the Western to Eastern Atlantic basins as well as throughout the Mediterranean Sea, where it may occur in sympatry with *O*. cf. *siamensis* [34] and *O. fattorussoi*. *Ostreopsis* cf. *ovata* also occurs in the Indo-Pacific region [9,14], where the genus *Ostreopsis* presents a high diversity. *Ostreopsis* cf. *ovata* should be considered a species complex [14,16] as it includes five morphologically identical but genetically distinct phylogroups, a few of them co-occurring at the *O. ovata* type locality (Ryukyu Islands, Japan), as shown by Sato et al. [14]. Geographic isolation and phylogenetic divergence [10,34] including the presence of Hemi Compensatory base changes (HCBCs) in the ITS2 region [9] suggest that *O*. cf. *ovata* from the Atlantic/Mediterranean/Pacific (AMP) sub-clade *sensu* Penna et al. [34] represents a separate taxonomic unit [34].

Molecular analysis of the ITS-5.8S regions, D1–D3 and D8–D10 regions of LSU rDNA confirmed that strains UNR-03, UNR-05, UNR-10 and UNR-60 isolated from different locations in Brazil as well as three field specimens isolated from Bahia can be assigned to *Ostreopsis* cf. *ovata*. All strains except UNR-10 grouped together in sub-clade A while strain UNR-10 grouped in a sister sub-clade (B) with two strains from the Madeira Archipelago at Portugal and one from Greece.

The presence of *O*. cf. *ovata* was registered by morphological identification at several places along the Brazilian coast and the oceanic islands (e.g., at Saint Pauls’ Rocks, Nascimento et al. [29]) within latitudes ranging from 27° 35′S to 05° 5′N. Molecular sequences of *O*. cf. *ovata* have been retrieved from Rio de Janeiro [10,26], Ubatuba, São Paulo [35], Salvador, Bahia [47], and Currais archipelago at Paraná [30]. The current study includes new molecular sequences from Mata de São João at Bahia, Tibaú do Sul at Rio Grande do Norte, and the oceanic archipelago of Fernando de Noronha. So far, *Ostreopsis* cf. *ovata* is the only *Ostreopsis* species registered in Brazil, however the coastline has been majorly under-sampled.

### 3.3. Toxins

The toxin profile determined for strains UNR-03 and UNR-05, as well as for bloom samples collected from Rio de Janeiro were similar and were composed mainly of ovatoxin-a (average 63.1%) and ovatoxin-b (average of 31.7%). In previous studies, OVTX-a and -b were dominant in the toxin profile of *O*. cf. *ovata* strains isolated from a nearby area in Rio de Janeiro [26], from Bahia [47] and from Currais Archipelago [30], all in Brazil. Dominance of OVTX-a and -b was also frequently reported in an extensive study assessing the toxin profiles of 55 *O*. cf. *ovata* strains along the Italian and French Mediterranean coast [48]. The toxin profile of *O*. cf. *ovata* from Rio de Janeiro was similar to that of 67% of the strains analysed by Tartaglione et al. [48] in the latter study. The toxicity data of ovatoxins is limited, as only ovatoxin-a has been isolated in a sufficient amount for full structure elucidation (the other OVTXs are minor congeners) and it is possible that different analogues may present different toxin potencies [48]. Considering the biological activity, OVTX-a hemolytic effect was shown to be lower than that of the reference PLTX compound, and a study using HaCaT cells viability indicated that OVTX-a is about 100-fold less potent than PLTX, but it is more toxic than Ostreocin-d (another PLTX analogue) [49].

Toxin cell quota (31.3 and 39.3 pg cell^−1^) was in the range of that reported for strains of *O*. cf. *ovata* from elsewhere [30,47,48]. In a previous study, two strains isolated from Rio de Janeiro showed higher toxin cell quotas, ranging from 60 to 468 pg cell^-1^, with the highest value measured from a senescent culture under poor growth [26]. High OVTX cell quotas were also observed in some strains from the Mediterranean Sea [50,51]. Several authors have shown that intracellular toxin content (cell quotas) can be variable between different strains and may vary for a single strain subjected to diverse environmental conditions and along the growth phase of a batch culture (e.g., Scalco et al. [38] and Pezzolesi et al. [52]).

At Armação dos Búzios, Rio de Janeiro, suspected cases of human intoxication occurred on 20 and 21 February 2014 when 60 people sought medical care after visiting Tartaruga beach. Beachgoers presented signs of respiratory and eye irritation, including conjunctivitis, nausea, and general malaise. There were anecdotal reports that a brownish stain floating at the sea surface was transported by the wind to the beach and that people felt sick after it reached the sand. On that occasion, Tartaruga beach was closed for a week but the causes of that intoxication were not identified. The symptoms described at that time were similar to those experienced by beach goers at the Mediterranean Sea during *O*. cf. *ovata* blooms [22,53]. However, samples collected on 22 February at Tartaruga beach, and on 26 February in a broader area close to that beach presented low (<10^3^ cells gFW^-1^macroalgae) abundances of *O*. cf. *ovata* (Nascimento, data not published). It might be possible that an *O*. cf. *ovata* bloom had detached from coastal islands nearby and cells were transported to the beach by the wind (as a “brownish stain”), but this is rather speculative.

## 4. Conclusions

Blooms of *O*. cf. *ovata* are common at Armação dos Búzios and Arraial do Cabo in Rio de Janeiro, Brazil, and the presence of moderate ovatoxin concentrations in bloom field samples highlights the potential risk these toxic proliferations may represent to marine fauna and human health in the area. The accumulation of ovatoxins in seafood harvested locally should be further studied. The presence of *O*. cf. *ovata* from the AMP (or A) sub-clade was confirmed at one oceanic island in the South Atlantic Ocean, and the occurrence of sub-clade B of *O*. cf. *ovata* was registered along the Brazilian Northeast coast. The variability in thecal plate morphology of *O*. cf. *ovata* observed by several authors was further confirmed in strains isolated from the South Atlantic Ocean.

## 5. Materials and Methods

### 5.1. Strains Isolations and Cultures Establishment

**Figure 8:**
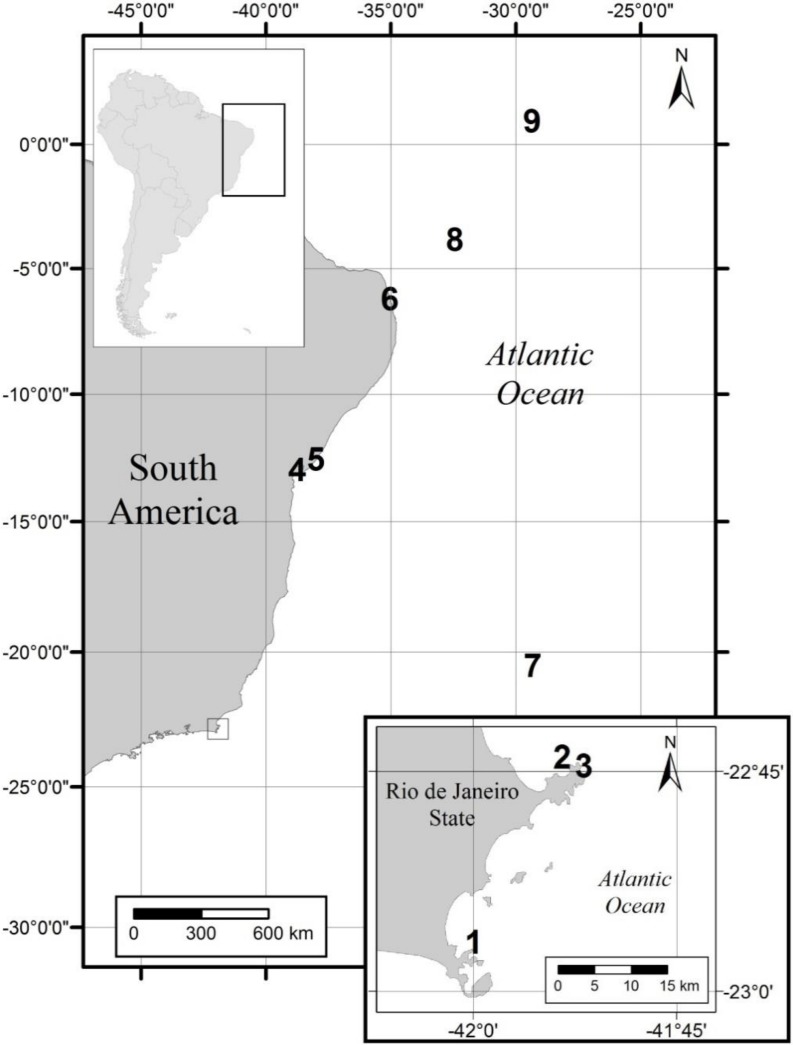
Map of the Brazilian coast showing the sites where samples have been collected for this study: 1. Forno, Arraial do Cabo, Rio de Janeiro (RJ); 2. Tartaruga, Armação dos Búzios, RJ; 3. Forno, Armação dos Búzios, RJ; 4. Penha, Bahia; 5. Forte, Bahia; 6. Mata de São João, Rio Grande do Norte; 7. Trindade Island; 8. Fernando de Noronha; 9. St. Paul´s Rocks. The inset shows the Rio de Janeiro state area in detail.

Macroalgal samples were collected from a depth of 1–2 m by snorkel diving from three locations in Brazil: Rio de Janeiro, Rio Grande do Norte and Fernando de Noronha Island (Figure 8, Supplementary Table S1) for the isolation of *Ostreopsis* strains. Specimens of macroalgae were placed in sealable plastic bags and vigorously shaken for 2 min to detach the associated epiphytic cells. Live cells of *Ostreopsis* were isolated from the epiphytic suspension using a micropipette and were sequentially transferred through four to five drops of sterile and filtered (glass-fiber filter, Millipore AP-40, Millipore, São Paulo, Brazil) local seawater. After each transfer, the drop was examined to ensure that only a single cell was present. After the final transfer, each isolated cell was placed into a separate well of a sterile 96-well tissue culture plate containing 120 µL of L2/2 culture medium [54] prepared with seawater which had been filtered (glass-fiber filter, Millipore AP-40, Millipore, São Paulo, Brazil), autoclaved, and the salinity adjusted to 34 with deionized water (dH_2_O). When sufficient cell density was achieved through successive cell division, cells were transferred to a separate well of a sterile 6-well tissue culture plate containing L2/2 medium and were eventually transferred to 250 mL glass Erlenmeyer flasks. All stock cultures were maintained in a temperature-controlled cabinet at 24 ± 2 °C, with a 12 h light:12 h dark cycle and a photon flux density of 60 µmol photon m^-2^ s^-1^ provided by cool-white fluorescent tubular lamps. Photosynthetically active radiation was measured with a QSL-100 quantum sensor (Biospherical Instruments, San Diego, CA, USA).

### 5.2. Morphological Characterization

Live cells or neutral Lugol iodine solution preserved cells of *Ostreopsis* cf. *ovata* strains UNR-03, UNR-05, UNR-10 were observed using a Leica DMLA light microscope (Leica Microsystems GmbH, Wetzlar, Germany) with phase contrast, differential interference contrast and epifluorescence optics, the latter using ultraviolet (UV) lamp HBO 100 W/2 (Osram GmbH, Munich, Germany) and a fluorescence filter cube with an excitation filter BP340-380 nm, a dichromatic filter 400 nm, and an emission LP 425 nm. For plate pattern identification, cells were stained with Fluorescent Brightener 28 (Sigma-Aldrich, St. Louis, MO, USA). Images were obtained using an AxioCam HRc digital camera and Zen image acquisition and analysis software (Zeiss, Oberkochen, Germany, version 1.1.1.0, 2012). Neutral Lugol iodine solution preserved cells of *Ostreopsis* cf. *ovata* strain UNR-60 were observed using an upright light microscope (ImagerA2, Zeiss, Germany) equipped with phase contrast. Images were obtained using an AxioCam MRc digital camera (Zeiss, Oberkochen, Germany) in this case. The dorso-ventral (DV) diameter (or depth) and width (W) of cells were measured in lugol preserved cells using the Axiovision software (Zeiss, Oberkochen, Germany). In this study, a modified Kofoid tabulation system [55] as described in Besada et al. [32] was followed to name the plates, and this enabled comparisons with other genera.

Additionally, the dorso-ventral diameter (DV), width (W), and DV/W of cells from field *Ostreopsis* cf. *ovata* populations were measured either using the Axiovision software (Zeiss, Oberkochen, Germany) or with a microscope eyepiece ruler. Morphometric analyses were performed on neutral Lugol iodine preserved cells present in epiphytic suspensions from macroalgae that have been collected from five sites along the Brazilian tropical and subtropical coast (Arraial do Cabo, Forno-Armação dos Búzios, Tartaruga-Armação dos Búzios, Forte-Bahia, Penha-Bahia) and from three oceanic islands (Fernando de Noronha, St Pauls´s Rocks, Trindade) at the South Atlantic (Figure 8, Supplementary Table S1) as described in Section 5.1.

Differences in the DV, W, and DV/W values among *O.* cf. *ovata* cells from three cultured strains (LCA-B7, LCA-E7, UNR-05) isolated from Rio de Janeiro, and eight surveyed field populations (Supplementary Table S1) were evaluated through a nonparametric Kruskal–Wallis test, followed by pairwise multiple comparisons (Wilcoxon matched-pairs test). Statistical analyses were performed using the software Statistica 8.0 (Statsoft). Graphs were plotted using the software GraphPad Prism 5.0 (GraphPad).

### 5.3. Molecular Characterization of Strains

Exponentially growing cells of *Ostreopsis* cf. *ovata* (strains UNR-03, UNR-05, UNR-10 and UNR-60) were harvested in 2 mL microtubes by centrifugation at 5000× *g* for 15 min for DNA extraction. The supernatant was discarded and the cell pellets were stored at −80 °C for further analysis. Genomic DNA was extracted from the pellets using the Qiagen DNeasy Plant Mini Kit (Qiagen Inc., USA) following the manufacturer’s instructions and then stored at −20 °C.

Three ribosomal DNA (rDNA) loci were analyzed in the present study: the D1–D3 and the D8–D10 regions of the large subunit (LSU); and the Internal Transcribed Spacer (ITS = ITS1-5.8S-ITS2). These *loci* have been successfully used in several phylogenetic studies of *Ostreopsis* species [9,14,15,16,33]. The D1–D3 region was amplified using the pair of primers D1R/LSUB (5′-ACCCGCTGAATTTAAGCATA-3′/5′-ACGAACGATTTGCACGTCAG-3′) [56,57]. The D8-D10 region was amplified using the pair of primers FD8/RB (5′-GGATTGGCTCTGAGGGTTGGG-3′/5′-GATAGGAAGAGCCGACATCGA-3′) [58]. The ITS region was amplified using the pair of primers ITSA/ITSB (5′-GTAACAAGGTHTCCGTAGGT–3′/5′-AKATGCTTAARTTCAGCRGG-3′) [14]. The amplification reaction mixture (25 μL) contained 1 unit (U) Taq DNA polymerase (Thermo Scientific Inc, USA), 1× reaction buffer with NH_4_SO_4_, 2.5 mM MgCl_2_, 0.16 mM dNTPs (Thermo Scientific, USA), 8 pmol of each primer and 15 ηg of genomic DNA. Reactions with LSU primers (D1–D3 and D8–D10) comprised an initial 5 min heating step at 94 °C, followed by 40 cycles at 94 °C for 1 min, 58 °C for 1 min, 72 °C for 1 min and a final extension at 72 °C for 5 min. Reactions with ITS primers comprised an initial 5 min heating step at 94 °C, followed by 40 cycles at 94 °C for 1 min, 45 °C for 1 min, 72 °C for 1 min, and a final extension at 72 °C for 5 min. The sequencing of samples was performed by Macrogen Inc. using both forward and reverse primers. Sequences were manually checked and edited using the software BioEdit v7.2.5 [59]. The new sequences obtained in the present study were BLAST-searched against the GenBank database (www.ncbi.nlm.nih.gov/blast) to test for sequence homology with non-target taxa. The sequences were then aligned with other *Ostreopsis* sequences retrieved from GenBank using MAFFT v7 [60]. Phylogenetic analyses were conducted separately for each molecular marker. Sequences of *Coolia* spp. were used as outgroup.

Amplified samples that did not result in good quality sequences were cloned in pGEM T-Easy vector^®^ (Promega), and the final constructs were introduced into DH5α cells. The colonies obtained were submitted to a colony PCR [61] according to conditions described above for the conventional PCRs.

The MEGA 7.0 software [62] was used to select the best-fit model of nucleotide substitution (GTR+G+I for D1–D3 and D8–D10; HKY+G+I for ITS) and to construct maximum likelihood (ML) phylogenetic trees with 1000 bootstrap (BS) replications. The phylogenetic relationships were also examined using Bayesian inference (BI) with MrBayes v3.2.6 [63]. The command “lset nst = mixed” was used before running the analysis in order to sample across nucleotide substitution models. Markov Chain Monte Carlo procedure consisted of two independent trials with four chains each. Each chain was run for 1 × 10^6^ generations and sampled every 100th cycle. Posterior probability (PB) values for the resulting 50% majority rule consensus tree were estimated after discarding the first 10% of trees as burn-in.

### 5.4. Molecular Characterization of Field Specimens

Single cells of *O.* cf. *ovata* were isolated from Lugol-preserved samples collected from the Forte site in Bahia (see Table S1), by micropipetting under Zeiss Primovert inverted light microscope (Zeiss, Oberkochen, Germany). Cells were rinsed into several drops of filtered and autoclaved seawater before transferring to a 0.2 mL PCR tube. PCR tubes were stored at −80 °C until direct PCR amplifications. Immediately before the PCR reactions the samples were submitted to the following heat shock procedure: 1 min at 95 °C followed by 1 min at 4 °C (ice). This procedure was repeated three times. Samples were then submitted to direct PCR reactions and sequencing analysis, using the ITS primers, as described for cultured samples above.

### 5.5. Toxin Analysis by Liquid Chromatography-Mass Spectrometry

Cultures of *O.* cf. *ovata* (strains UNR-03 and UNR-05) grown (as described in Section 5.1) in 125 mL Erlenmeyer flasks containing modified L2/2 medium were harvested for toxin determinations. The volume of each culture was measured and split into two 50 mL centrifuge tubes. A sub-sample of 3 mL was removed from each centrifuge tube and preserved with neutral Lugol iodine solution for cell enumeration using a Sedgewick-Rafter chamber. Cultures were harvested by centrifugation for 15 min (5000× *g*) to settle the cells into pellets. The supernatant was discarded and the cell pellets were stored at −80 °C and then freeze dried for toxin analysis.

The biofilm of an *Ostreopsis* cf. *ovata* bloom covering macroalgae at Rio de Janeiro was collected using a 50 mL glass syringe for toxin analysis at two occasions (see Table S1). These bloom samples were collected on May 21st 2012 from Forno beach, Arraial do Cabo and from Tartaruga beach at Armação dos Búzios on December 8th 2014 for toxin qualitative analysis as cell enumeration was not performed in these samples. Bloom samples were transferred to 50 mL centrifuge tubes and transported to the laboratory in a cool box. Once in the laboratory, bloom samples were harvested by centrifugation at 5000× *g* for 15 min to settle the cells into pellets. The supernatant was discarded and the cell pellets were stored at −80 °C.

Toxins were extracted from *Ostreopsis* cf. *ovata* cultivated or field-sampled cells by adding 90% methanol in a proportion of 1 mL for 1 million cells and disrupting the cells under bath ultrasound (Transonic TI-H-15, Elma^®^, Germany) at 45 kHz for 15 min. Extracts were centrifuged at 1200 x *g* for 15 min, and the supernatant was filtered (NanoSep 0.2-µm Nylon filter, PALL^®^, UK). Toxin determination was achieved by liquid chromatography coupled to tandem mass spectrometry (LC-MS/MS) on a Shimadzu^®^ LC system (UFLC-XR, Japan) connected to a hybrid triple quadrupole/ion-trap mass spectrometer (API 4000 QTrap, ABSciex^®^, USA), as described in Tibiriçá et al. [30]. Three multiple reaction monitoring (MRM) transitions were monitored in positive ionization mode for each of the following compounds: m/z 1324.2→327.2, 1315.2→327.2 and 877.2→327.2 for OVTX-a; 1346.3→371.2, 1337.3→371.2 and 891.8→327.2 for OVT-b; 1354.3→371.2, 1345.3→371.2 and 897.2→327.2 for OVTX-c; 1332.2→327.2, 1323.2→327.2 and 882.5→327.2 for OVTX-d; 1332.2→343.2, 1323.2→343.2 and 882.5→343.2 for OVTX-e; 1338.3→327.2, 1329.3→327.2 and 886.5→327.2 for OVTX-f; and 1340.2→327.2, 1331.2→327.2 and 887.8→327.2 for PLTX. All toxins were quantified using a calibration curve made of serial dilutions of the PLTX standard (Wako Chemicals GmbH, Neuss), assuming equal molar response, and expressed as PLTX equivalent (PLTX-eq.). Resulting limits of detection (LOD) and quantification (LOQ) were estimated to be 20 and 40 ng PLTX-eq. mL^−1^, respectively.

## Figures and Tables

**Table 1:** Dorso-ventral (DV) diameter and width (W) in µm, and DV/W ratio of cultured and field cells of *Ostreopsis* cf. *ovata* from diverse locations showing range of dimensions and mean ± standard deviations values in brackets.

Sample Location	DV	W	DV/W	Origin	Reference
Rio Grande do Norte, Brazil (strain UNR–10)	47.4–64.0 (53.7 ± 5.7)	37.4–57.7 (42.3 ± 7.8)	1.1–1.4 (1.3 ± 0.1)	Culture	Current study
Fernando de Noronha Island, Brazil	30.0–57.5 (45.1 ± 6.6)	17.5–37.5 (26.3 ± 4.8)	1.3–2.3 (1.7 ± 0.2)	Field	Current study
Forte, Bahia, Brazil	36.6–59.5 (49.0 ± 5.2)	21.0–42.6 (29.3 ± 4.5)	1.4–2.1 (1.7 ± 0.1)	Field	Current study
Penha, Bahia, Brazil	48.4–85.1 (63.7 ± 7.3)	31.3–60.7 (45.7 ± 6.1)	1.2–1.7 (1.4 ± 0.1)	Field	Current study
Trindade Island, Brazil	30.0–82.5 (58.7 ± 11.2)	12.5–58.8 (29.0 ± 9.2)	1.2–3.2 (2.1 ± 0.4)	Field	Current study
Armação de Búzios, Rio de Janeiro, Brazil	39.2–65.0 (53.6 ± 6.2)	24.1–58.8 (31.8 ± 5.8)	0.9–2.0 (1.7 ± 0.2)	Field	Current study
Armação de Búzios, Rio de Janeiro, Brazil	28.6–62.3 (49.7 ± 6.0)	16.6–41.6 (29.8 ± 4.9)	1.4–2.2 (1.7 ± 0.1)	Field	Current study
Armação de Búzios, Rio de Janeiro, Brazil (strain UNR–05)	29.8–61.4 (45.0 ± 6.4)	21.1–45.2 (32.5 ± 5.1)	1.2–1.7 (1.4 ± 0.1)	Culture	Current study
Arraial do Cabo, Rio de Janeiro, Brazil (strain LCA–B7)	33.0–68.0 (53.0 ± 10.8)	18.0–45.0 (31.1 ± 8.2)	1.4–2.5 (1.7 ± 0.2)	Culture	Nascimento et al. [26]
Arraial do Cabo, Rio de Janeiro, Brazil (strain LCAE7)	40.0–62.0 (52.6 ± 5.8)	20.0–48.0 (34.4 ± 6.1)	1.2–2.0 (1.6 ± 0.2)	Culture	Nascimento et al. [26]
Arraial do Cabo, Rio de Janeiro, Brazil	40.0–65.0 (51.3 ± 7.5)	18.0–45.0 (28.3 ± 6.7)	1.4–2.5 (1.9 ± 0.3)	Field	Nascimento et al. [26]
Saint Paul’s Rocks, Brazil	45.9–65.6 (55.4 ± 4.1)	27.5–45.6 (35.7 ± 3.9)	1.3–1.9 (1.6 ± 0.1)	Field	Nascimento et al. [29]
Currais Archipelago, Paraná, Brazil (strains LM062, LM086, LM129, LM130)	23.7–60.1 (40.8 ± 8.7)	15.4–48.9 (31 ± 7.5)	1.04–1.68 (1.33 ± 0.13)	Culture	Tibiriçá et al. [30]
Currais Archipelago, Paraná, Brazil	29.9–65.9 (49.9 ± 6.6)	17.1–45.9 (32.6 ± 5.9	1.31–1.79 (1.53 ± 0.12)	Field	Tibiriçá et al. [30]
Ubatuba, São Paulo, Brazil	35–65 (55.1)	20–40 (32.6)	(1.69)	Field	Gómez et al. [35]
Hainan Island, China (strains 1S1D2, 1S1D4, 1S1D6)	39.9–56.4 (47.5 ± 3.1)	30.4–47.4 (37.1 ± 3.3)	1.1–1.4 (1.3 ± 0.1)	Culture	Zhang et al. [33]
Gulf of Gabès, Mediterranean Sea (strains Oso.1, Oso.2, Oso.3, Oso.4, Oso.5, Oso.6, Oso.7)	27–65	19–57	–	Culture	Abdennadher et al. [36]
Heron Island, Australia (strain HER27)	30.2–48.3 (37.7 ± 4.3)	21.9–37.5 (28.7 ± 3.7)	1.1–1.8 (1.3)	Culture	Verma et al. [9]
Ebro Delta, Spain, NW Mediterranean Sea	21.24–76.9 (54.50 ± 6.80)	15.57–51.0 (33.05 ± 5.51)	–	Field	Carnicer et al. [37]
Iberian Peninsula, Spain, Atlantic Ocean	54.8–84.3 (69.6 ± 7)	30.3–62.0 (44.7 ± 6.3)	1.2–1.9 (1.6 ± 0.1)	Field	David et al. [12]
Gulf of Naples, Tyrrhenian Sea, Mediterranean (strain D483)	(49.5 ± 5.1)	(33.3 ± 4.7)	–	Culture	Scalco et al. [38]
Portonovo, Adriatic Sea, Mediterranean (strain CBA–T)	(49.2 ± 4.1)	(33.7 ± 4.8)	–	Culture	Scalco et al. [38]
Gulf of Trieste, Adriatic Sea, Mediterranean (strain OS2T)	(46.8 ± 6.1)	(32.8 ± 5.4)	–	Culture	Scalco et al. [38]
Conero Riviera, Adriatic Sea, Mediterranean	18.7–75.0	12.5–60.0	–	Field	Accoroni et al. [39]
Ussuriiskii Bay (Peter the Great Bay) Sea of Japan	36–60 (49.4 ± 6.4)	24–45 (29.6 ± 5.3)	–	Field	Selina and Levchenko [40]
Gulf of Trieste, Italy, Adriatic Sea, Mediterranean	48–65	31–46	1.33–1.74	Field	Honsell et al. [41]
Subogata, Otsuki Town, Kochi, Japan (strain s0726)	(28.1 ± 2.6)	(21.2 ± 2.8)	–	Culture	Sato et al. [14]
Gulf of Trieste, Italy, Adriatic Sea, Mediterranean	29.6–70.8 (55.3 ± 8.0)	18.5–53.1 (36.4 ± 6.4)	–	Field	Monti et al. [42]
Rovinj, Croatia, Adriatic Sea, Mediterranean	33.3–66.6 (54.8 ± 7.1)	18.5–44.4 (34.3 ± 4.7)	–	Field	Monti et al. [42]
North Aegen Sea, Mediterranean	26.2–61.9	13.1–47.6	–	Field	Aligizaki and Nikolaidis [43]
Port Dickson, Malaysia (strains OvPD04, OvPD06,OvPD07)	33–41	24–34	–	Culture	Leaw et al. [44]
Kota Kinabalu, Malaysia (strains OvSA02, OvSA04, OvSA06, OvSA09, OvSA10)	32–55	22–39	–	Culture	Leaw et al. [44]
Pulau Redang, Malaysia (strains OvPR01, OvPR02, OvPR03, OvPR04)	44–48	33–37	–	Culture	Leaw et al. [44]
Rangaunu Harbour, New Zealand	38–50	25–35	–	Field	Chang et al. [45]
Civitavecchia, Italy, Tyrrhenian Sea, Mediterranean	34.2–66.6 (55.7 ± 6.1)	25.2–39.6 (31.9 ± 4.1)	–	Field	Tognetto et al. [46]
French Polynesia, New Caledonia and Ryukyu Islands	50–56	25–35	–	Field	Fukuyo [2]

**Table 2:** Ovatoxin profile (% of total concentration) and intracellular quotas (in brackets, in pg cell^−1^) of strains UNR-03 and UNR-05 and from two bloom samples, collected from Armação dos Búzios and Arraial do Cabo, Rio de Janeiro state, Brazil. Cell quotas were not determined for bloom samples.

Samples	OVTX-a	OVTX-b	OVTX-c	OVTX-d	OVTX-e
Strain UNR-03	58.5 (20.9)	40.0 (14.3)	0.5 (0.2)	0.4 (0.1)	0.6 (0.2)
Strain UNR-05	68.2 (20.0)	31.7 (9.3)	0.03 (<0.1)	0.02 (<0.1)	0.05 (<0.1)
Armação dos Búzios bloom	61.0	26.7	4.1	2.5	5.7
Arraial do Cabo bloom	64.7	28.3	1.3	2.2	3.4

## Supplementary Materials

The following are available online at https://www.mdpi.com/2072-6651/12/2/70/s1, Table S1. Summary of samples analysed in the current study, indicating the nature of each sample, site of origin, date collection and the analysis performed in each one, Figure S1: Size variability in dorso-ventral diameter (DV) of cultivated (LCA-E7, LCA-B7, UNR-05) and field cells of *O*. cf. *ovata* from coastal locations at Rio de Janeiro state (Arraial do Cabo, Forno, Tartaruga) and Bahia (Forte, Penha) and from the oceanic islands of Fernando de Noronha, St. Pauls’s Rocks and Trindade, Brazil.
