# Supplementary material for: Ostreopsis cf. ovata (Dinophyceae) Molecular Phylogeny, Morphology, and Detection of Ovatoxins in Strains and Field Samples from Brazil
Source: Toxins (Basel). 2020 Jan 22;12(2):70. doi: 10.3390/toxins12020070 (PMC7076807; doi:10.3390/toxins12020070)
Supplement: Supplementary file 1 [file toxins-12-00070-s001.pdf]

Supplementary Materials: *Ostreopsis* cf. *ovata* (Dinophyceae) Molecular Phylogeny, Morphology, and Detection of Ovatoxins in Strains and Field Samples from Brazil

Silvia M. Nascimento, Raquel A. F. Neves, Gabriela De'Carli, Geovanna T. Borsato, Rodrigo A. F. da Silva, Guilherme A. Melo, Agatha M. de Morais, Thais C. Cockell, Santiago Fraga, Adriana D. Menezes-Salgueiro, Luiz L. Mafra Jr, Philipp Hess and Fabiano Salgueiro

Table S1. Summary of samples analysed in the current study, indicating the nature of each sample, site of origin, date of collection and the analysis performed in each one.

Sample	Place of origin	Date	Molecular analysis	Thecal plates observation	Morpho metrics	Toxins
strain UNR-03	Tartaruga, Armação dos Búzios RJ (22°45′18′′ S, 41°54′07′′ W)	October 2012	x	x		x
strain UNR-05	Forno, Armação dos Búzios, RJ (22°45′42″S, 41°52′34″ W)	February 2013	x	x		x
strain UNR-10	Rio Grande do Norte (6°13′34″S 35°02′58″W)	September 2013	x	x		
strain UNR-60	Fernando de Noronha (3°52′33″S 32°28′04″W)	October 2016	x	x		
O. cf. *ovata* single cell 1	Forte, Bahia (12º34′41″ S, 38º00′04″ W)	January 2013	x	x		
O. cf. *ovata* single cell 2	Forte, Bahia (12º34′41″ S, 38º00′04″ W)	January 2013	x	x		
O. cf. *ovata* single cell 3	Forte, Bahia (12º34′41.0″ S, 38º00′04.7″ W)	January 2013	x	x		
Bloom 1	Forno, Arraial do Cabo, RJ (22º 59′ S, 42º 00′W)	May 2012				x
Bloom 2	Tartaruga, Armação dos Búzios (22°45′18′′ S, 41°54′07′′ W)	December 2014				x
Field	Forno, Arraial do Cabo, RJ (22º 59′ S, 42º 00′W)	December 2006			x	
Field	Tartaruga,Armação dos Búzios, RJ (22°45′18′′ S, 41°54′07′′ W)	February 2012			x	
Field	Forno, Armação dos Búzios, RJ (22°45′42″S, 41°52′34″W)	December 2012			x	
Field	Penha, Bahia (12º59′27″ S, 38º37′29″W)	July 2012			x	
Field	Forte, Bahia (12º34′41″ S, 38º00′04″W)	January 2013			x	
Field	Trindade Island (20º29′22″ S, 29º20′04″ W)	September 2014			x	
Field	Fernando de Noronha (3°52′33″S 32°28′04″W)	October 2017 & 2018			x	
Field	Saint Paul's Rocks (0°55′10″N 29°20′33″W)	July 2009			x	

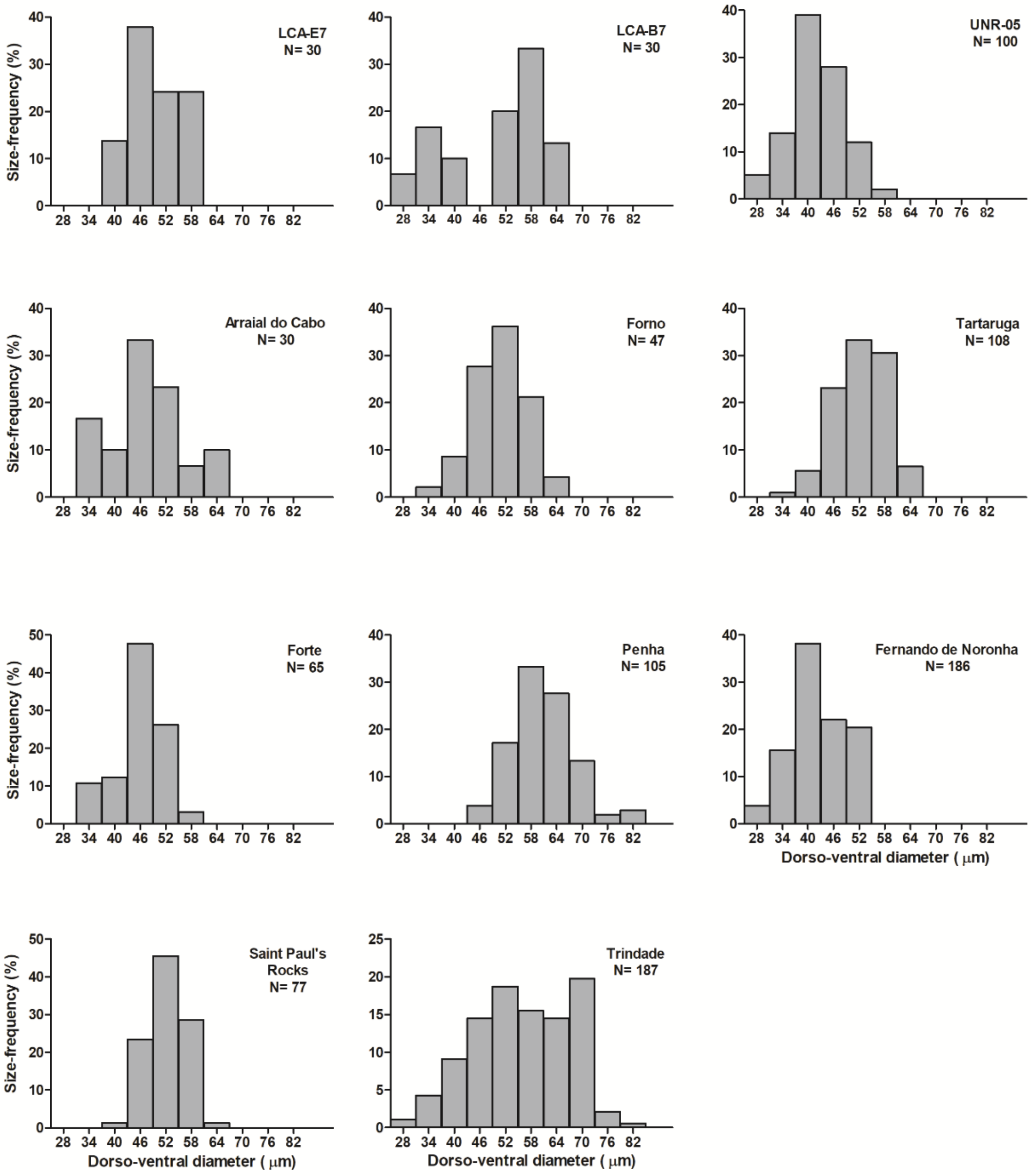

Figure S1. Size variability in dorso-ventral diameter (DV) of cultivated (LCA-E7, LCA-B7, UNR-05) and field cells of *O.* cf. *ovata* from coastal locations at Rio de Janeiro state (Arraial do Cabo, Forno, Tartaruga) and Bahia (Forte, Penha) and from the oceanic islands of Fernando de Noronha, St. Pauls's Rocks and Trindade, Brazil.
